# SNP discovery of Korean short day onion inbred lines using double digest restriction site-associated DNA sequencing

**DOI:** 10.1371/journal.pone.0201229

**Published:** 2018-08-07

**Authors:** Ji-Hee Lee, Sathishkumar Natarajan, Manosh Kumar Biswas, Kenta Shirasawa, Sachiko Isobe, Hoy-Taek Kim, Jong-In Park, Chi-Nam Seong, Ill-Sup Nou

**Affiliations:** 1 Department of Horticulture, Sunchon National University, Suncheon, Jeonnam, South Korea; 2 Department of Biology, Sunchon National University, Suncheon, Jeonnam, South Korea; 3 Kazusa DNA Research Institute, Kisarazu, Chiba, Japan; 4 University-Industry Cooperation Foundation, Sunchon National University, Suncheon, Jeonnam, South Korea; Youngstown State University, UNITED STATES

## Abstract

Onion (*Allium cepa* L.) is an economically important vegetable crop around the world. Genetic and genomic research into various onion accessions will provide insights into the onion genome to enhance breeding strategies and improve crops. However, the onion’s large genome size means that studies of molecular markers are limited in onion. This study aimed to discover high quality single nucleotide polymorphisms (SNPs) from 192 onion inbred lines relating to short-day cultivation in Korea. Paired-end (PE) double digested restriction site-associated DNA sequencing (ddRAD-seq) was used to discover SNPs in onion. A total of 538,973,706 reads (25.9 GB), with an average of 2,658,491 high-quality reads, were generated using ddRAD-seq. With stringent filtering, 1904 SNPs were discovered based on onion reference scaffolds. Further, population structure and genetic relationship studies suggested that two well-differentiated sub-populations exist in onion lines. SNP-associated flanking sequences were also compared with a public non-redundant database for gene ontology and pathway analysis. To our knowledge, this is the first report to identify high-quality SNPs in onion based on reference sequences using the ddRAD-seq platform. The SNP markers identified will be useful for breeders and the research community to deepen their understanding, enhance breeding programs, and support the management of onion genomic resources.

## Introduction

Onion (*Allium cepa* L., 2n = 16) is an important monocotyledonous crop that is widely cultivated and consumed worldwide. It belongs to the *Amaryllidaceae* family, which also includes garlic, shallots, and scallions. These crops are grown in temperate and tropical regions, highly valued for culinary purposes. They inhabits nutritional, medicinal and pharmacological benefits, including being anticarcinogenic, anti-inflammatory, antimicrobial, and antifungal [1–3]. China and India, where 65.5% of the world’s human population live, are the leading producers of onions, producing large quantity every year (89 million tons in 2014, 91 million tons in 2015, and 92 million tons in 2016 [Food and Agriculture Organization, http://faostat.fao.org]). Most onions grown in Korea are short-day and open-pollinated while the annual production of onions is affected by several biotic and abiotic components of the ecosystem [4,5]. Most onion producers have noted decreased yields caused by bacterial (e.g., Brown Rot, *Pseudomonas aeruginosa*), viral (e.g., Aster yellows), and fungal diseases (e.g., Purple Blotch, *Alternaria porri*; *Stemphylium* leaf blight (SLB), *Stemphylium vesicarium*; White Rot, *Sclerotium cepivorum Berk*; Basal Rot, *Fusarium oxysporum* f.sp.; Downy Mildew, *Peronospora destructor*; Onion Smut, *Urocystis cepulae*; Onion Smudge, *Colletotrichum circinans*; Black Mould, *Aspergillus niger*; and Neck rot, *Botrytis allii*). Environmental stresses (abiotic factors) such as high temperature, salinity, drought, and soil nitrogen deficiency also limit onion production and quality.

Currently, available genomic information about diploid alliums is limited and the fact that the main genome database AlliumMap is not publically/freely accessible. The complex genome of onions, in particular with a complexity of 16.3 Gb per 1C nucleus have created technical difficulty in the development of molecular markers [6]. Although onions are highly valuable vegetable crops with pharmacological benefits, data about their genetic and genomic makeup remain limited. Extensive genetic and genomic research must be conducted to further understand the onion genome to enhance crop improvement and adaptation, develop accessions that are resilient to biotic and abiotic stresses, and increase onion quality and quantity. DNA-based molecular markers have been extensively used to accelerate plant breeding programs through marker-assisted selection for improving germplasm efficiency, and to understand the molecular mechanisms underlying genetic traits. Numerous genetic markers, including simple sequence repeats (SSRs) [7], expressed sequence tag SSRs (EST SSRs) [8], Inter-simple sequence repeats (ISSRs) [9], amplified fragment length polymorphisms (AFLPs) [10], randomly amplified polymorphic DNA (RAPD) [11] and single nucleotide polymorphisms (SNPs) [12] have been developed and used to determine genetic diversity, construct genetic linkage maps, and conduct phylogenetic analyses of onion germplasm [13]. SNPs are considered to be the most reliable genetic markers, with advantages of flexibility, cost-effectiveness, rapid, and low error rate [14]. SNP markers can easily be converted to perform high-throughput assays, and to support onion breeding programs with existing technologic resources. In recent decades, genome-wide SNP discovery has been accelerated in several plant and animal species [15] with the aid of next generation sequencing (NGS) technology [16].

To date, the most recently developed genotyping methods are genotyping by sequencing (GBS) [17] and restriction site-associated DNA sequencing (RAD-seq) These simple, techniques reduce the complexity of large and multifarious genomes for easier genome-wide SNP discovery, and have been used in several plant species including onion inbred lines [12], garlic [18], maize [19], barley and wheat [19], and soybean [20]. They are also cost-effective ways of performing high-throughput sequencing of large sample sets in a single experiment, and offer the possibility of detecting SNPs on a large scale, with or without reference genome sequence. Recently, Shirasawa established a ddRAD-Seq (double-digest restriction-site-associated DNA sequencing) workflow to sequence the genotypes of complex genomes with higher accuracy than GBS [21].

In this study, we used paired-end (PE) ddRAD-seq technology to develop a novel reference-based genome-wide SNP resource from onion inbred lines cultivated in Korea. Filtered high-quality SNPs from 192 cultivars related to short-day inbred lines were subjected to population structure and genetic relationship studies. In addition, we functionally annotated SNP flanking sequences to determine similarity with known genes and biological functions.

## Materials and methods

### Plant materials and DNA extraction

The 192 short-day onion inbred lines used for this study were purchased from four Korean companies: Nonghyup Seed (NH: 40), Bio Energy Crop Research Institute (Muan) (B: 39), Changnyeong Onion Research Institute (CN: 36), and Nongwoo Seed (NW: 77) (Table 1 and S1 Table). To extract genomic DNA (gDNA), fresh young leaves from plants of each inbred line were collected, immediately frozen in liquid nitrogen, and stored at –80°C until further use. Total genomic DNA was isolated using the Qiagen DNeasy Plant Mini Kit (Qiagen, Hilden, Germany) according to the manufacturer’s standard protocol. The quality and quantity of isolated DNA samples was measured with 1% agarose gel electrophoresis, and a Nanodrop spectrophotometer (Thermo Scientific, Delaware City, DE, USA), respectively. Samples were diluted to 50 ng/μL for ddRAD sequencing.

**Table 1:** The information of 192 Korean onion inbreds used in this study.

S.No	Number of accessions	Sample type	Cultivation	Company	Country
**1**	40	Inbred lines	Short day	Nonghyup Seed company^a^	Korea
**2**	39	Inbred lines	Short day	Bio Energy Crop Research Institute (Muan)^b^	Korea
**3**	36	Inbred lines	Short day	Changnyeong Onion Research Institute^c^	Korea
**4**	77	Inbred lines	Short day	Nongwoo Seed Company^d^	Korea

^a^ Nonghyup Seed: http://nhseed.nonghyup.com/

^b^ Bio Energy Crop Research Institute (Muan): http://www.nics.go.kr

^c^ Changnyeong Onion Research Institute: http://cnonion.or.kr/

^d^ Nongwoo Seed Company: http://www.nongwoobio.co.kr

### Double digest restriction site associated (ddRAD) DNA sequencing

Genomic DNA from each line was double-digested with PstI and EcoRI restriction enzymes. ddRAD-Seq libraries were constructed using two combinations of restriction enzymes, and the sequencing procedure published by Shirasawa *et al*. [21] was followed. Adaptor-ligated DNA amplicons were pooled and separated using 1.5% agarose gel electrophoresis by BluePippin (Sage Science, Beverly, MA, USA). DNA fragments with lengths of 300–900 bp were isolated using the QIAGEN MiniElute Gel Extraction Kit (Qiagen). Finally, constructed ddRAD-seq libraries were sequenced using the HiSeq platform (Illumina, USA), using the 93-bp PE mode for each inbred line.

### ddRAD- seq analysis and SNP detection

Sequencing data obtained from 192 inbreds were examined for their quality using the FastQC tool (http://www.bioinformatics.babraham.ac.uk/projects/fastqc/). Low-quality sequences were removed using PRINSEQ (http://prinseq.sourceforge.net) [22] and adaptor sequences were removed using fastx_clipper from the FASTX-Toolkit (version 0.10.1; http://hannonlab.cshl.edu/fastx_toolkit). A total of 8,822,891 cleaned and filtered sequence reads of five libraries for B001, CNH001, CNJ001, NH001, and NW001 were assembled with “large or complex genome” mode of Newbler v3.0 (Roche). The resultant 12,718 sequences spanning 1,599,536 bases were used as a reference for the following analysis. The reads from the PE sequences of each accession were mapped to the reference sequence using Bowtie 2 (version 2.1.0) [23]. The resulting sequence alignment/map format (SAM) files were converted to Binary Alignment/Map (BAM) files, and SAMtools (version 0.1.19) was used to sort, index and remove duplicates [24]. Genomic variants (SNPs) were called out for each onion lines against the reference genome using the mpileup module from SAMtools and the BCFtools view option. Variant call format (VCF) files produced, including SNP details, were further filtered with a SNP quality score of ≥ 999, minimum depth of 5, minor allele frequency of 0.05, and minimum proportion of missing data of 0.5 for each locus using VCFtools (version 0.1.11) [25]. Missing data were imputed using Beagle4 [26], and the filtered high-confidence SNPs from ddRAD-Seq were subjected to further analysis.

### Population structure analysis

The heterozygosity and the percentage polymorphic loci was calculated in the onion inbreds using GenAlex version6.03. The population structure of the Korean onion lines was estimated using STRUCTURE (version 2.3.4) [27] with data from detected SNPs. This program uses a model-based Bayesian clustering algorithm approach to correlate allele frequencies for independent runs without the need for population information. Ten independent runs were performed with different *K* values from 1–10 (*K* is the number of distinct, strong differentiations between genetic groups and unknowns). For this, the Markov Chain Monte Carlo (MCMC) length of the burn-in period was set at 30,000 iterations, and after a burn-in period, the number of iterations was adjusted to 50,000 steps. The admixture model was implemented to obtain the optimal *K* value. We followed a delta-*K* procedure based on the method published by Evanno et al. [28], using the online program STRUCTURE Harvester (web version 0.6.94; http://taylor0.biology.ucla.edu/structureHarvester/) [29] to estimate the optimal *K* value from independent runs. The population structure comprising SNPs detected from inbred lines were visualized using STRUCTURE with the following options: (i) select optimal *K* value run, (ii) show plot as ‘bar plots’, and (iii) sort by Q [30].

### Genetic relationship analysis

GenAlEx software (version 6.5) was used to calculate pairwise relatedness (genetic distance) between inbred lines, and principal component analysis (PCA) was performed using TASSEL (version 5.2.42) [31]. The generated pairwise distance matrix file was used to construct a phylogenetic tree for population differentiation. The MEGAv7 [32] program was used to generate a neighbor-joining tree, with bootstrap values based on genetic distance matrices with default settings.

### Functional analysis of SNP-associated scaffolds

SNP-associated scaffold sequences were retrieved from the reference genome and used as BLASTX queries against the non-redundant protein database at the National Center for Biotechnology Information (NCBI). BLAST parameters were as follows: e-value cut-off, 1.0E-5; word size, 3; number of BLAST hits, 3; and other parameters, default. The most similar sequence matches for each SNP-associated scaffold was selected based on multiple hits, and these were used to find Gene Ontology (GO) terms, and enzyme and pathway details using Blast2GO suite (http://www.blast2go.com/b2ghome). The three major GO terms, biological process (BP), cellular component (CC), and molecular function (MF), were determined with annotation cut-offs of ≥ 55; GO weight, 5; and e-value hit filter, < 1.0E-6 [33]. Details of enzymes and pathways were searched for using the ‘Enzyme Code and KEGG’ option in Blast2GO, and data was retrieved from the KEGG (Kyoto Encyclopedia of Genes and Genomes) database [34].

## Results and discussion

### ddRAD- seq data analysis and SNP discovery

Genomic DNA isolated from 192 onion inbred lines was used to prepare ddRAD (PstI and EcoRI) representation libraries. These constructed libraries were then successfully sequenced using the Illumina HiSeq platform. PE sequencing of individuals yielded 538,973,706 reads, with an average of 2,807,154 (~2.8 million) reads per accession, covering 25.9 GB of sequenced data. An average of 2,658,491 high quality reads were obtained and used for reference genome alignment. The reference genome comprised 12,718 scaffolds, with an average scaffold length of 126 bp (range: 96–556 bp). An average of 33.3% reads were aligned to the onion reference genome, with the reference genome alignment ratio ranging between 17.2% and 51.5%. ddRAD-sequenced accessions contained 37.7% GC content on average (range 36–43%). A statistical summary of data collected about raw reads, cleaned reads, reference genome-mapped reads, and alignment ratios for individual accessions are summarized in S2 Table. In addition, 192 PE raw reads were deposited in NCBI sequence read archive (SRA) with accession SRP150117.

**Fig 1:**
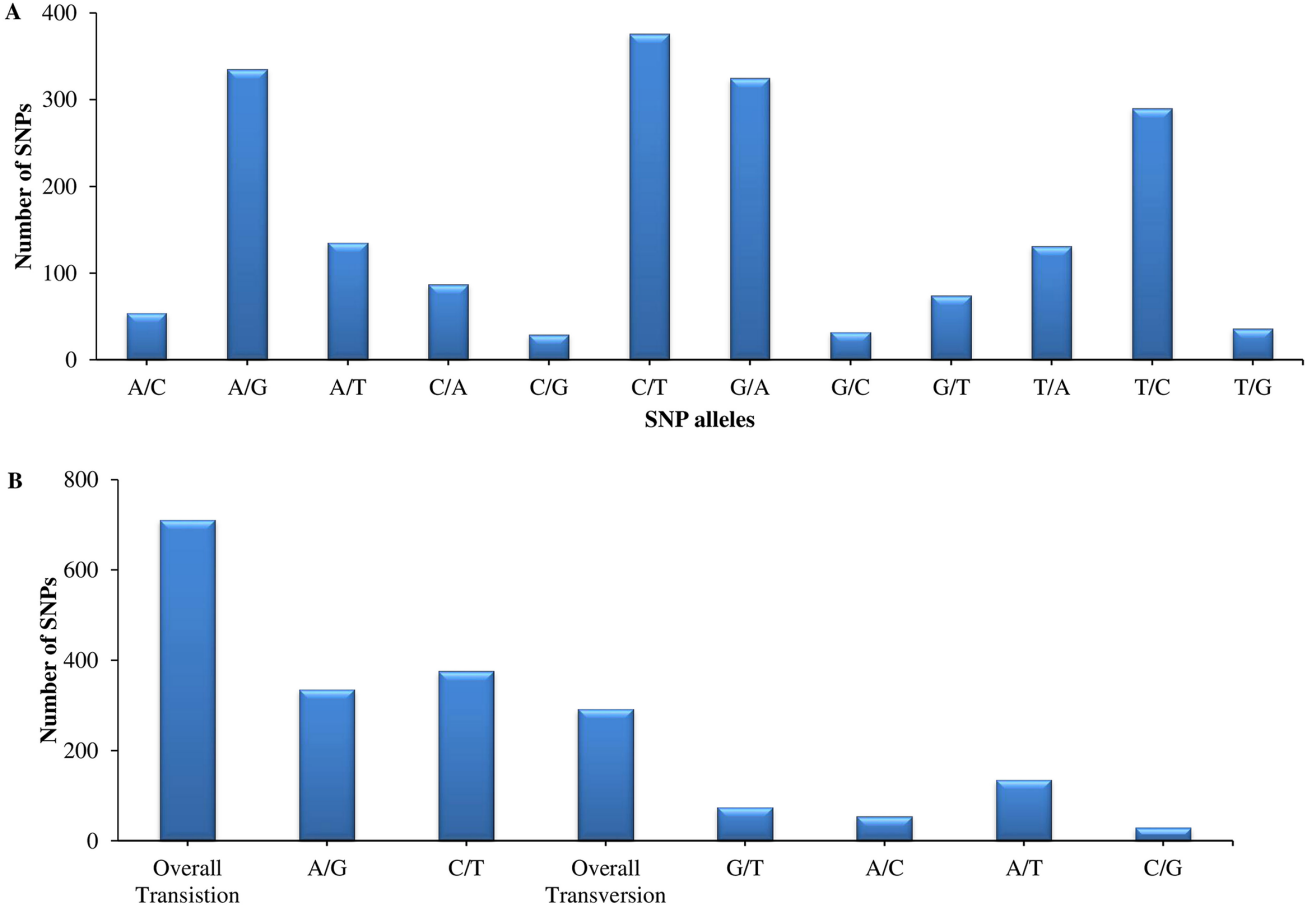
Plots showing SNP distribution (A), and transition/transversion ratios (B) for SNPs identified from ddRAD-sequencing.

Mapped reads were further investigated to identify SNPs. A total of 1904 SNPs were identified from ddRAD-sequences of all 192 onion inbred line accessions, and these SNPs comprised 558 scaffolds (S3 Table). High-quality SNPs were filtered based on a SNP quality score of ≥999, minimum depth of 5, minor allele frequency of 0.05, and minimum proportion of missing data of 0.5 using the VCFtools program. Distributions of each type of SNP were as follows: C/A, 87 (4%); G/A, 325 (17%); G/C, 32 (2%); T/A, 131 (7%); T/C, 290 (15%) and T/G, 36 (2%) (Fig 1A). Of the 1904 identified SNPs, 711 (38%) were classified as transitions (A/G or C/T), and 292 (15%) were classified as transversions (G/T, A/C, A/T, or C/G) (Fig 1B). In general, transitions occurred more frequently than transversions because of the interchange between purine and pyrimidine nucleotide bases. In addition, to estimate real sequencing data, a transition/transversion ratio of >0.5 was used. This ratio was used to calculate divergence and to restructure the phylogenetic tree [35,36]. The C/T allele occurred most frequently (376; 20%) among SNP alleles, which is a consistent observation in *Allium cepa* [37], and similar to findings in other species including C*ucumis melo* [38], *Brassica napus* [39], and oil palm [40]. The transition/transversion ratio in this study was 2.53, which is lower than has been previously reported in wheat (1.75) [41], similar to that observed in rice (2.3) [42], and higher than observed in peanut (3.2) [43]. A genetic map of Korean inbred lines was constructed using the GBS method without a reference genome [12]. However, with sufficient SNP flanking regions, reference sequence (contig/scaffolds)-based RAD-sequencing data analysis was successfully used to design a SNP array and construct high density genetic/linkage maps [44–46]. Therefore, the resulted SNPs with associated flanking sequences might be useful for high-throughput validation assays in onion breeding programs for crop improvement.

### Population structure and genetic relationship analysis

The heterozygosity and the percentage of polymorphic loci calculated using GenAlex (version 6.5) among the four populations genotyped using 1,904 SNPs showed that the observed heterozygosity (*H*_*o*_) is less than the expected heterozygosity (*H*_*e*_). The mean *H*_*o*_ and *H*_*e*_ of the onion inbred lines among the four populations were calculated as 0.00 and 0.36, respectively (S4 Table). The relative low observed heterozygosity than expected frequency shows the influence of inbreeding force in these onion lines selected for study. In maize inbred lines, a similar effect was reported with *H*_*o*_ < *H*_*e*_ [47]. In addition, the highest percentage of polymorphic loci was found in the onion populations of Nongwoo Seed accessions. The mean percentage of polymorphic loci observed among the four populations were found to be 97.93 revealing high diversity in their SNP genotypes due to inbreeding.

**Fig 2:**
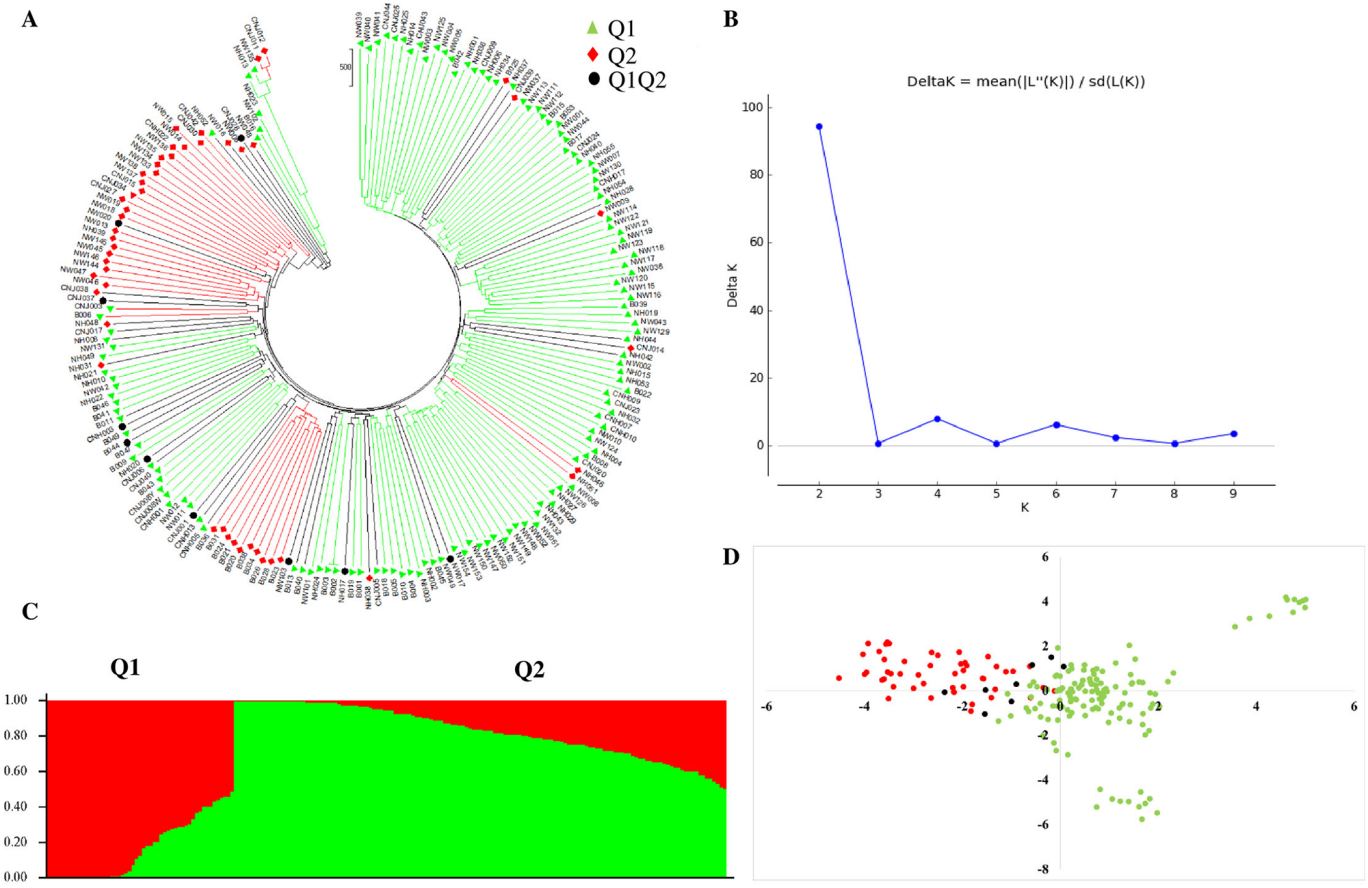
Model-based population structure analysis of 192 Korean onion accessions. (A) Neighbor-joining phylogenetic tree using MEGA 7 (color codes based on population structure); (B) delta-K values from STRUCTURE Harvester using the Evanno method; (C) two population structure classifications from 192 onion accessions using the STRUCTURE program; (D) principal component analysis of the first two components. The color codes (Q1 [cluster 1], red; Q2 [cluster 2], green) of each onion accession were consistent in A and C based on population structure analysis.

To investigate the genetic relationships between 1904 SNPs from Korean onion inbred lines, a phylogenetic tree was constructed based on a pairwise distance matrix using neighbor-joining methods (Fig 2A). The 192 lines were classified into three main clades based on clustered SNPs: clade 1 contained 44 accessions, clade 2 contained 48, and clade 3 contained 100. Each main clade was further classified into subclades: clade 1 contained two subclades with 29 and 15 accessions, and clade 2 contained a further two subclades with 37 and 11 accessions. The largest clade, clade 3, contained two major subclades with 60 and 40 accessions, and other accessions were grouped together.

Using the STRUCTURE 2.3.4 program, a model-based clustering approach was used to analyze the population structure of 192 Korean onion inbred lines. The optimal delta-*K* value was determined using STRUCTURE Harvester, which revealed the highest delta-*K* value to be *K* = 2 (Fig 2B), suggesting that two well-differentiated sub-populations exist within these accessions. Population structure for each accession was plotted using Sort Q. The STRUCTURE program used a cut-off value of 0.55 for clustering based on genotype information. As expected, Korean onion inbred lines were distributed into two different clusters or populations, represented by standard color codes used in this program: Q1 (cluster 1) red, and Q2 (cluster 2) green. In addition, mixture of Korean accessions contained both Q1 and Q2 color codes. Q1 accounted for 49 (25.52%) inbred accessions, and Q2 accounted for 135 (70.31%) inbred accessions. The remaining 8 (4.16%) accessions contained a mixture of Q1 and Q2.

These results also correlated with the phylogenetic tree, where red (Q1), green (Q2), and blue (mixture of Q1/Q2) colors can be seen (Fig 2C). Phylogenetic tree analysis revealed that that 192 accessions are clearly divided into three clades, consistent with the results from STRUCTURE. Principal component analysis (PCA) based on a two-dimensional distribution in TASSEL (Fig 2D) was also consistent with the population structure and neighbor-joined cluster analyses.

In practice, breeders preferred to select plant materials based on their germplasm collections, relatedness limitations and long term consistent assistance to support breeding programs. Relatedness analysis is important for this purpose, and helps breeders to understanding the backgrounds of their plant materials. This model can also be used to obtain results for genomic selection and association-related studies in various plant species such as large garlic (*Allium sativum*) [18], cowpea [48], and others [49].

### Functional analysis of SNP-associated scaffolds

**Fig 3:**
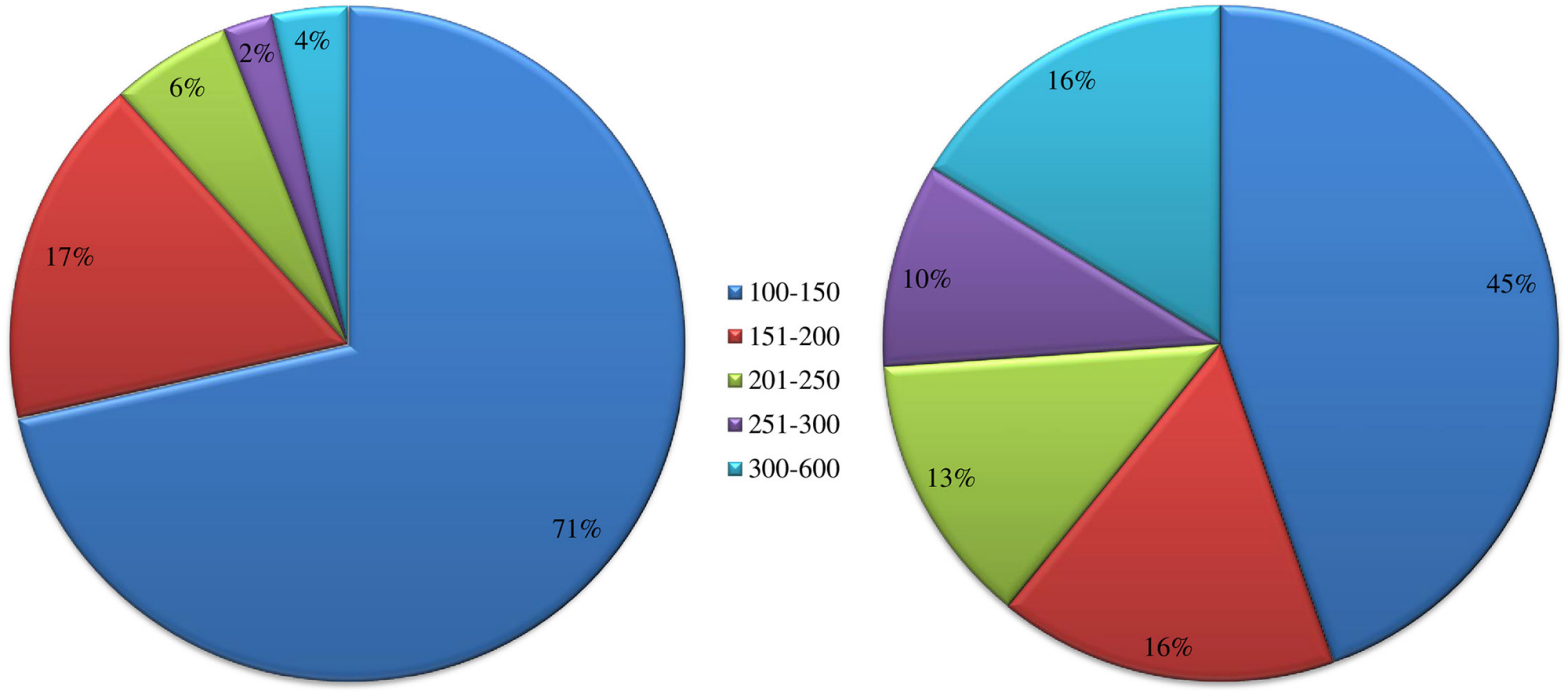
Sequence length distribution from the reference genome (left), and number of sequences annotated with BLAST hits (right).

**Fig 4:**
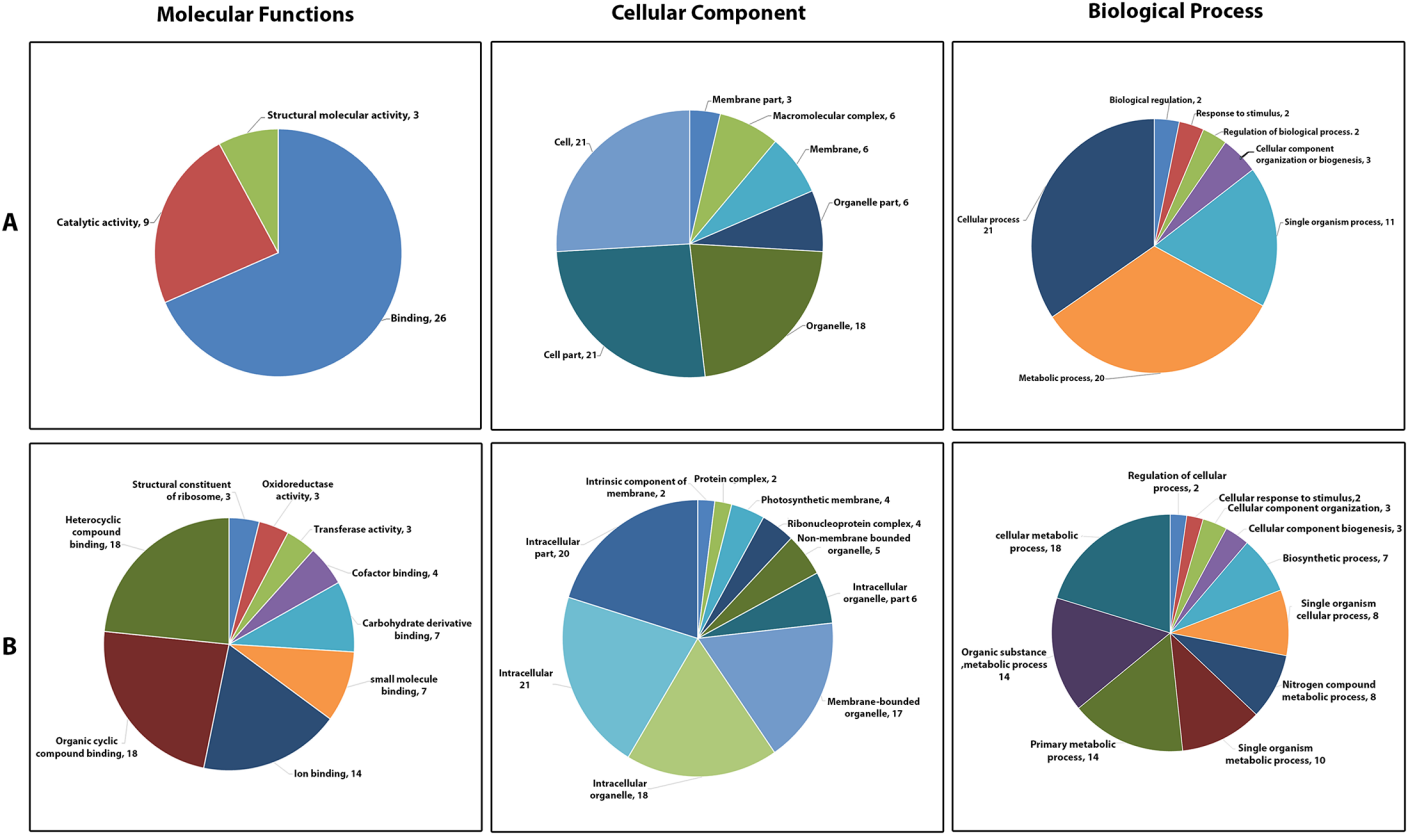
Level 2 (A) and level 3 (B) Gene Ontology classifications of the SNP-associated scaffolds identified from ddRAD-sequencing.

A total of 558 SNP-associated scaffolds were blasted against the NCBI non-redundant protein sequence database using BLASTX via Blast2GO. BLAST similarity results obtained 92 hits from 558 scaffolds corresponding to known protein sequences (E-value <1.0E-5) (S5 Table). The remaining 466 scaffolds did not match with any known protein sequences from a public database, suggesting that our SNP-associated scaffold sequences were unique to Korean onion inbred lines (Fig 3) [37]. The 92 BLAST hits mainly matched with *Asparagus officinalis*, Allium species (*Allium cepa*, *Allium fistulosum*, and *Allium microdictyon*), and *Daucus carota subsp*. *sativus*. In addition, functional annotations resulted in 78 GO tserms with 41 blast hits. These 78 GO terms were further classified into three functional categories such as cellular component (CC; 22 GO terms), molecular function (MF; 33 GO terms), and biological process (BP; 23 GO terms). Some scaffolds matched with more than one GO term, whereas a few matched only one GO term (S6 Table). Cellular component annotations were further subclassified into seven major level predominant GO subcategories; cell (GO: 0005623) and cell part (GO: 0044464) categories were associated with 21 scaffolds; organelle (GO: 0043226), 18 scaffolds; organelle part (GO: 0044422) 6 scaffolds; membrane (GO: 0016020), 6 scaffolds; membrane part (GO: 0044425) 3 scaffolds; and macromolecular complex (GO: 0032991) 6 scaffolds (Fig 4A). Most scaffolds in the molecular function categories were associated with binding (GO: 0005488; 26 scaffolds), catalytic activity (GO: 0003824; 11 scaffolds), and structural molecule activity (GO: 0005198; 3 scaffolds).

Biological process GO terms were also categorized into seven subcategories: cellular process (GO: 0009987), 21 scaffolds; metabolic process (GO: 0008152), 20 scaffolds; response to stimulus (GO:0050896) 2 scaffolds; cellular component organization or biogenesis (GO:0071840) 3 scaffolds; biological regulation (GO:0065007) 2 scaffolds; regulation of biological process (GO:0050789) 2 scaffolds; and signaling (GO:0023052), 1 scaffold. Detailed classification of level 3 GO terms are plotted in Fig 4B.

The number of annotated scaffolds discovered in this study is less than those in known genome sequences; this finding is similar to previous studies that have conducted de-novo transcriptome analysis. In addition, these kind of results obtained due to the sequence lengths, and depth SNP or scaffold coverage mean these results might be unique to Korean onion inbred lines [37]. Analysis of pathway details from annotation results shows that 7 scaffolds are involved in 12 different pathways (Table 2). Of note, scaffold06984 consists of 1 SNP (46: T/C; position, SNP allele) that is involved in three pathways: cysteine and methionine metabolism (map00270), biosynthesis of antibiotics (map01130), and sulfur metabolism (map00920). Further, scaffold00480 (4: A/T), scaffold01554 (37: C/G; 38: G/T; 43: C/T; 53: A/G, and 64: C/T), and scaffold01794 (111: T/A) are involved in nucleotide synthesis metabolism (purine metabolism, pyrimidine metabolism and thiamine metabolism), and nucleotide sugar metabolism (amino sugar and nucleotide sugar metabolism, galactose metabolism). Finally, 3 scaffolds, scaffold00116 (58: A/G), scaffold00052 (92: A/G), and scaffold00012 (106: G/C, 169: T/C, and 322: C/T) were identified in the oxidative phosphorylation (map00190) pathway. The above-mentioned homology search, along with species distribution, annotation and pathway details from RAD-sequencing, will provide valuable resources for understanding more about short-day Korean onion inbred lines.

**Table 2:** Pathway details of annotated SNP-associated scaffolds.

Pathway ID	KEGG pathway	Number of sequences	Enzyme
map00270	Cysteine and methionine metabolism	1	O-acetyltransferase [ec:2.3.1.30]
map00052	Galactose metabolism	1	Phosphatase [EC:3.6.1.15], RNA polymerase [EC:2.7.7.6]
map00920	Sulfur metabolism	1	Dehydrogenase [EC:1.6.99.3]; reductase (H+-translocating) [EC:1.6.5.3]
map00520	Amino sugar and nucleotide sugar metabolism	1	4-Epimerase [EC:5.1.3.2]
map00240	Pyrimidine metabolism	1	O-Acetyltransferase [EC:2.3.1.30]
map01130	Biosynthesis of antibiotics	1	4-Epimerase [EC:5.1.3.2]
map00730	Thiamine metabolism	1	RNA polymerase [EC:2.7.7.6]
map00230	Purine metabolism	2	O-Acetyltransferase [EC:2.3.1.30]
map00190	Oxidative phosphorylation	6	Phosphatase [EC:3.6.1.15]

## Conclusions

We identified highly valuable SNP resources from Korean onion lines using ddRAD-seq analysis. To our knowledge, this is the first report of reference scaffolds being used for the discovery of SNPs related to short-day cultivation in Korean onion lines. The high-quality SNPS identified from this study, with details of their genetic makeup and functional annotations, will be useful for deepening our understanding and updating our knowledge of onion genomic resources. Furthermore, markers developed from the SNPs we have found might be used for onion breeding programs, cultivar identification, marker-assisted selection programs, and high-density map development and validation with high throughput sequencing methods in future.

## Supporting information

S1 Table List of the 192 Korean onion inbred accessions, sample codes, sample types, resource, and locations assigned in this study. (XLSX)

S2 Table List of 1,904 SNPs identified in Korean onion inbreds. (XLSX)

S3 Table Statistical summary of raw reads, filtered reads, and reference genome alignment of each Korean onion inbred individual from ddRAD-sequencing. (XLSX)

S4 Table Allele frequency and heterozygosity measurements among the 192 onion inbreds. (DOC)

S5 Table BLAST results of SNP-associated sequences from Korean onion accessions compared with the non-redundant (nr) protein database. (XLSX)

S6 Table Gene Ontology (GO) annotations of Korean onion accessions. (XLSX)
