# Supplementary material for: SNP discovery of Korean short day onion inbred lines using double digest restriction site-associated DNA sequencing
Source: PLoS One. 2018 Aug 7;13(8):e0201229. doi: 10.1371/journal.pone.0201229 (PMC6080773; doi:10.1371/journal.pone.0201229)
Supplement: S4 Table — (DOC) [file pone.0201229.s004.doc]

**Table S4. Allele Frequency and Heterozygosity measurements among the 192 onion inbreds**

| **Population** | ***N*** | ***Na*** | ***Ne*** | **Mean Observed Heterozygosity**  **(*H_o_*)** | **Mean Expected Heterozygosity**  **(*H_e_*)** | **Polymorphic Loci (%)** |
| --- | --- | --- | --- | --- | --- | --- |
| **Nonghyup Seed company** | 29.981 | 2.576 | 1.688 | 0.000 | 0.363 | 97.53 |
| **Bio Energy Crop Research Institute (Muan)** | 25.330 | 2.569 | 1.730 | 0.000 | 0.374 | 97.79 |
| **Changnyeong Onion Research Institute** | 28.386 | 2.571 | 1.702 | 0.000 | 0.368 | 97.85 |
| **Nongwoo Seed Company** | 54.110 | 2.699 | 1.679 | 0.000 | 0.362 | 98.53 |
| **Mean** | **34.452** | **2.604** | **1.700** | **0.000** | **0.367** | **97.93** |
| ***N* = Number of Alleles**  ***Na* = Number of Different Alleles**  ***Ne* = Number of Effective Alleles** | | | | | | |
